# The Role of Toll-Like Receptor in Inflammation and Tumor Immunity

**DOI:** 10.3389/fphar.2018.00878

**Published:** 2018-08-06

**Authors:** Xiaohong Cen, Shuwen Liu, Kui Cheng

**Affiliations:** Guangdong Provincial Key Laboratory of New Drug Screening, Guangzhou Key Laboratory of Drug Research for Emerging Virus Prevention and Treatment, School of Pharmaceutical Sciences, Southern Medical University, Guangzhou, China

**Keywords:** Toll-like receptors, tumor microenvironment, programmed cell death, tumor immunotherapy, immune adjuvant

## Abstract

Toll-like receptors (TLRs) activation enables host to recognize a large number of pathogen-associated molecule patterns (PAMPs), ignite immune cells to discriminate between self and non-self, and then promote the following innate and adaptive immune responses. Accumulated clinical/preclinical evidences have proven TLRs to be critical role in the autoimmune diseases, including inflammatory and tumor-associated diseases. Activation of TLRs is becoming or has been a target for cancer treatment. It is shown that TLRs can induce preferable anti-tumor effect by eliciting inflammatory cytokines expression and cytotoxic T lymphocytes (CTLs) response. As adjuvant, TLRs agonists can launch a strong immune response to assist cancer radiotherapy and bio-chemotherapy. On the other hand, tumor-associated antigens acting as PAMPs, can also activate TLRs and induce tumor gene-related programmed cell death, including apoptosis, autophagy and programmed necrosis. While there are also arguments that the excessive TLRs expression will promote tumor deterioration in various organisms, as the TLR-induced inflammation will accelerate the cancer cells boost in the tumor microenvironment (TME). However, the effect of TLRs acting on cancers is still not quite clear today. In this review, we will summarize the recent researches of TLRs in cancer treatment and their role in TME, giving a brief overview on future expectation.

## Introduction

As the best-characterized class of pattern recognized receptors (PRRs), most endosomal and plasma-membrane associated Toll-like receptors (TLRs) have been found to respond to conserved microbial products and endogenous damaged molecules such as lipopolysaccharide (LPS), lipopeptides, flagellin, bacterial DNA, viral double-stranded RNA (dsRNA), as well as high mobility group box 1 (HMGB1) and beta-defensins (O’Neill et al., 2009), which constitute the first-line of organismal defense against invading microbial pathogens, tissue injury or cancer, playing an important role in innate immune response and the subsequent of adaptive immune response (Kawai and Akira, 2011). TLRs have attracted a substantial of interest in cancer research due to the clinical relevance of TLRs expression and tumor control (Pradere et al., 2014). Accumulated evidences indicate that artificial activation of TLRs on the immune cells, including monocytes, macrophages, and dendritic cells (DCs), can generate strong efficacy to boost therapy-elicited anticancer immunity, characterized by priming CD8+ T-cell and natural killer cells (NK cells) (Mogensen, 2009; Cheadle et al., 2017).

While TLRs are expressed not only in immune cells, but also tumor microenvironment (TME) (Gonzalez-Reyes et al., 2010). TME is a complex arrangement of cancer cells, normal cells, stromal tissue and extracellular cytokine. Stromal cells, the major components of TME, including cancer-associated fibroblasts (CAFs), tumor-associated macrophages (TAMs), marrow-derived suppressive cells (MDSCs), and regulatory T cells (Tregs) (Sato et al., 2009; Grivennikov et al., 2010). TLRs expressed among TME can not only induce self-programmed cell death, but also release cytokines and chemokine in the tumor environment, recruiting the immune cells to further release pro-inflammatory cytokines, pro-angiogenic factors and growth factors (Killeen et al., 2006; Zhou et al., 2009), such as TGFβ, IL-8, CXCR4, ICAM-1, and VEGF, which may repair the anti-tumor function of antigen-presenting cells (APCs) and effector T-cells as well as apoptosis response. This inappropriate immune enhancement and anti-tumor immune through TLRs signaling pathway, will act as signal transducers to control tumor progression, metastasis, recurrence and chemotherapy tolerance (Connolly and O’Neill, 2012).

At present, tumor-antigen specific immune response and gene-regulated death are the two main strategies on cancer elimination by activate various signaling pathways and adaptor proteins. To better understand the complex interaction of anti-tumor effect through TLRs signaling pathway, we cover the recent advances of TLRs and cancer in this review, mainly according immunotherapy and cancer cells programmed death, hoping to make a contribution in novel strategies for variety of cancers.

## TLRs and Programmed Cell Death

### Programmed Cell Death

**FIGURE 1:**
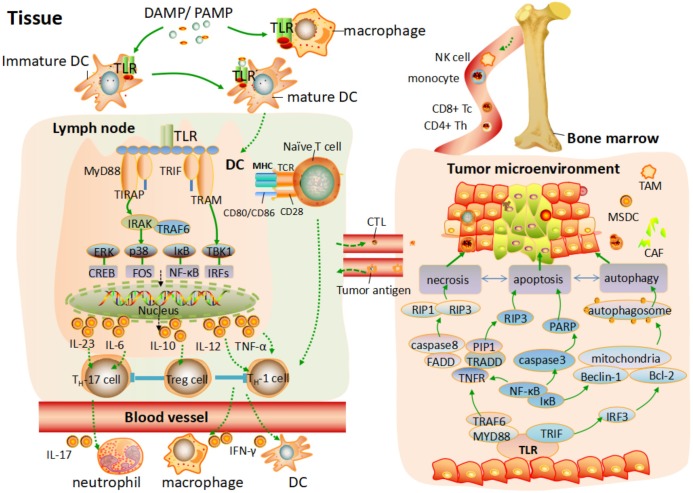
TLRs mediated the T cells response and programmed cell death process toward tumor environment. After TLRs bind to PAMPs or DAMPs, the activation of pro-inflammatory response and different programmed cell death process are elicited. On one hand, various antigen-presenting cells (APC) are activated, and a programmed maturation is initiated to induce the migration of APC to lymph node. Different TLRs are activated by MYD88 and TRIF to elicit the downstream pathway, leading to the phosphorylation of NF-κB or IRFs and consequent transcription of various genes coding pro-inflammatory cytokine. This cytokine production response to PAMPs/DAMPs and activates different subset of T cells. In addition, naive T cells in the lymph node activate. Differentiated T cells migrate through the blood to the tissue or tumor environment and are further activated by different factor. On the other hand, tumor gene-related programmed cell death is elicited by activation of TLRs signaling pathway. Apoptosis, autophagy, and necrosis are independent but have certain association with each other, which share the same signaling pathways or downstream effected adaptor to induce cancer cell death.

Programmed cell death (PCD), systematically classified into apoptosis, autophagy and programmed necrosis, is proposed to be cell dying in a pathological state to keep host in equilibrium via specific cellular mechanism and various signaling pathways (Tan et al., 2009). Apoptosis, autophagy and programmed necrosis can be distinguished by their special morphological differences and physiological process (Zhivotovsky, 2011). All of them have certain association with each other and they may share the same signaling pathways or downstream effected adaptors to induce cancer cells death. It is suggested that apoptosis and programmed necrosis are the two manners of tumor cell death, while autophagy is contributed to both tumor cell death and survival (Ou et al., 2017). Molecules released from damaged issue, involving pathogens and cells toxic components are named damage-associated molecular patterns (DAMPs), which can be recognized by different TLRs to trigger various cellular response. Tumor-associated antigens released in the tumor environment, can act as DAMP to activate TLRs signaling pathway or downstream adaptor proteins, initiating different mechanism of PCD toward various cancer cells (Ouyang et al., 2012). Here, we give a brief of how TLRs are involved into the tumor cell death by different mechanism PCD and provide a guide for better tumor therapy. The intrinsic relation of TLRs and PCD toward tumor is showed in **Figure 1**.

### TLRs Induce Apoptosis in Tumor Cells

Apoptosis, a gene controlled cell death to maintain internal environment homeostasis, is the most common type of PCD. Multiple evident have shown the correlation of TLRs and apoptosis that stimulation of hepatocellular carcinoma (HCC) with poly(I:C) (**Table 1**) can promote apoptosis by activation of TLR3 (Yoneda et al., 2008). Flagellin, a TLR5 agonist, was also found to induce HeLa cells death (Hancz et al., 2018). Mechanically, apoptosis conducted by TLRs is mainly divided into extrinsic and intrinsic way. The extrinsic pathway mediated the TNF receptor superfamily by TLR adaptor protein MYD88. It recruits the Fas-associated death domain (FADD) and pro-caspase-8 to autocatalytic activation of caspase-8, leading the proteolysis of pro-caspase-3 into active caspase-3 and triggering the apoptotic process (Meier and Vousden, 2007; Koizumi et al., 2016). On the other hand, under the control of mitochondrial pro-enzymes, the intrinsic pathway is to promote internal cytochrome c release into cytosol from the permeable mitochondrial membranes, and subsequently recruit Apaf-1 and pro-caspase-9, which will activate the downstream caspase-9/3 signaling cascade and then result in apoptosis (Ou et al., 2017). In addition, several studies have also demonstrated that TLRs are involved into different mechanism to induce apoptosis as well. For example, Imiquimod (IMQ) (**Table 1**), a synthetic nucleotide-like TLR 7 ligand, which has suggested to simultaneously induce ROS production to stimulate ATM/ATR pathway, leads to p53-dependent apoptosis in a skin basal cell carcinoma cell line (Huang et al., 2015). It is currently used as a topical and non-invasive treatment for superficial basal cell carcinoma (BCC), viral warts and other skin lesions in the clinic (Novak et al., 2008). In addition, TLRs can also disrupt the matrix metalloproteinase (MMP) to broke electron transfer chain and ATP synthesis, releasing the pro-apoptotic molecules to cytoplasm and consequently inducing tumor cells apoptosis (Reshma et al., 2016).

**Table 1:** Ongoing clinical study of TLRs agonists in cancer treatment as adjuvant.

Compound	Target	Combination	Indication	Clinical phage	Reference
Poly ICLC	TLR3	Radiotherapy	Cutaneous T-cell lymphoma	Phase I	NCT02061449
		NY-ESO-1 protein	Melanoma	Phase I/II	NCT01079741
		autologous tumor lysate vaccine	Anaplastic astrocytoma	Phase I	NCT01204684
		rhuFlt3L/CDX-301	Low-grade B-cell lymphoma	Phase I/II	NCT01976585
		Autologous OC-L Vaccine	Ovarian cancer	Phase I	NCT02452775
GLA-SE	TLR4	Radiotherapy	Stage III/ IV Adult soft tissue sarcoma	Phase I	NCT02180698
		MART-1 Antigen	II-IV Melanoma	Phase I	NCT02320305
G100	TLR4	Pembrolizumab	Follicular non-hodgkin’s lymphoma	Phase I/II	NCT02501473
Imiquimod	TLR7	Radiotherapy	Stage III or stage IV melanoma	Phase I	NCT00453050
		Cyclophosphamide	Breast cancer	Phase I/II	NCT01421017
Resiquimod (R848)	TLR7/8	NY-ESO-1 protein	Melanoma	Phase I	NCT00821652
		MAGE-3	Melanoma	Phase I	NCT00960752
		Peptide vaccine (LPV7)	Melanoma	Phase I/II	NCT02126579
MEDI9197	TLR 7/8	durvalumab	Solid tumors	Phase I	NCT02556463
VTX-2337	TLR8	Pegylated liposomal doxorubicin (PLD)	Ovarian cancer	Phase I	NCT01666444
		Cyclophosphamide	Solid tumors	Phase I	NCT02650635
		Cetuximab	Squamous cell cancer of head and neck	Phase I	NCT01334177
		Radiotherapy	Low-grade B-cell lymphoma	Phase I/II	NCT01289210
MGN1703	TLR9	Ipilimumab	Advanced solid malignancies	Phase I	NCT02668770
CPG 7909	TLR9	Radiotherapy	Non-hodgkin lymphoma	Phase I	NCT00453050
SD-101	TLR9	Ipilimumab	Low-grade B-cell lymphoma	PhaseI/II	NCT02254772
		Pembrolizumab	Prostatic neoplasms	Phase I	NCT03007732
		Ibrutinib	Grade 1–3A follicular lymphoma	PhaseI/II	NCT02927964
		Anti-OX40 antibody BMS	Low-grade B-cell lymphoma	Phase I	NCT03410901
		Radiotherapy	Low-grade B-cell lymphoma	Phase I/II	NCT02254772
EMD 1201081	TLR9	5-FU + Cisplatin + cetuximab	Squamous cell cancer of head and neck	Phase I	NCT01360827

*Data derived from http://clinicaltrials.gov.*

### TLRs Induce Autophagy in Tumor Cells

Autophagy is characterized with the formation of double-membrane-bound structure autophagosomes which warp the cancer cell or tumor-associated components with lysosomes and then degrade the contents in the capsule (Lin et al., 2013). Evident shows that autophagy are interconnected with apoptosis, as autophagy can either stimulate apoptosis by depleting tumor-associated suppressor, or impair tumor cells apoptosis by selectively reducing pro-apoptotic proteins, exhibiting dual effect on tumor control (Colell et al., 2007). The instinct of autophagy to remove the damaged proteins are crucial important to the last desperate efforts for survival of tumor cells. Various TLRs are proposed as autophagy inductor, which activate autophagy process by the downstream signaling adaptor MYD88 or TRIF, recruiting the TRAF6, and Beclin-1 to activate and generate autophagosomes (Shi and Kehrl, 2010). It is showed that IMQ (**Table 1**) can not only induce apoptosis in BCC, but also contribute to autophagic cell death in melanoma cell lines (Huang et al., 2015; Chang et al., 2017). However, Zhan et al. suggested that autophagy process induced by TLRs such as TLR3 and TLR4, will also facilitate migration and invasion of lung cancer cells by release various harmful cytokines such as IL6, CCL2, and MMP2 (Zhan et al., 2014). In addition, the patients with higher TLR4 expression are showed more LC3II (a maker of autophagosomes) in their cancer associated fibroblasts (CAFs), which is in proportion to more aggressive relapse rate and poorer prognosis in 180 luminal breast patients (Zhao et al., 2017). To be noted that, for the dual effect of autophagy, the cancer treatment with TLRs agonists on autophagy cell death need a integrate consideration in clinical studies.

### TLRs Induce Programmed Necrosis in Tumor Cells

Unlike apoptosis in a caspase-dependent manner, programmed necrosis is another mechanistically distinct cell death by phosphorylation and formation of RIP3 and PIR1 in a caspase-independent manner. In brief, necrosis, characterized by tremendous inflammatory response in cancer cells, enable to lyse mitochondrial and disrupt lysosomal membrane, leading to cell lysis or organelle dysfunction, which is crucial to maintain host in regular function (Cho et al., 2009). The study of the mechanism underlying TLR-mediated programmed necrosis is not quite clear. Nevertheless, TLR3 are proposed to induce necrosis partially dependent on TNF downstream signaling pathway and the subsequently to activate RIP adaptor protein to generate of RIP1 and RIP3 (Bianchi et al., 2017). It is also suggested that mouse colon carcinoma CT26 cells treated with poly(I:C) (**Table 1**) can not only induce an immune response, but also induce necrosis toward tumor cells exhibiting increased RIP1 and RIP3 interacting protein by TLR3/TICAM1-reactive oxygen species (Takaki et al., 2017). It shows that the underlying reaction between necrosis and TLRs are just beginning, more profound mystery is remain to explore.

## TLRs and Immune System

### TLRs Promote the Activation of Cytokines and Antitumor CTL Response

Toll-like receptors play an important role in the innate immunity for the ability to recognize various molecular products derived from pathogens and endogenous molecules releases from cancer or dying cells (Vigneron, 2015), and subsequently boost immune response. In brief, when various TLRs on the immune cells bind to specific ligands, APCs such as macrophages and DCs are activated (Pulendran et al., 1999). Then, a programmed of maturation is initiated to induce the migration of APCs into lymph node and the accumulated cytokines such as TNFα, IL6, IL12, MHC, and IFNs are subsequently released, which is crucial to the activation of NK cells and various cytotoxic T lymphocytes (CTLs) (Kopp and Medzhitov, 2003; Obermajer et al., 2018). For example, the lack of IFN-α in the tumor environment, deficits the ability of DC toward nucleic acids of tumor, leading to the immune tolerance and aggravation of cancer (Intidhar et al., 2012). While CpG ODN, an oligonucleotides agonists to TLR9, is reported to mediate TRIF downstream pathway to activate the IRF3 and IRF7, and then subsequently induce IFN-α and IFN-β (Combes et al., 2017).

However, generation of T cells is an extremely complex process. CD80 and CD86, including in the B7 superfamily, are vital in activating T cells to produce cytokines and generate CTLs, while CD40 is essential in the priming phase to stimulate DC, which enable DCs migrate to secondary lymphoid organs (Sharpe and Freeman, 2002). T cell activation occurs only after the interaction of T cell receptor (TCR) with tumor-associated antigen-MHC complexes in the addition of CD28 costimulation with CD80 and CD86 (Sharma and Allison, 2015). Up regulation of adhesive and Co-stimulatory molecules on APC is required. Studies showed that activation of TLR4 can initiate the downstream pathway to increase the expression of CD80, CD86, CD40, and IL-12, which can promote the process of T cells (Clarke, 2000). Of note that, IL-12 can also play an important role in eliciting antigen-presenting process in CTLs, which skew the effector from an established Th2 to a Th1 response in immune cells (Heufler et al., 1996).

On the other hand, although CTLs can recognize and kill tumor cells to maintain the equilibrium of internal environment, playing a significant role in the tumor immunosurveillance, the CD4+ CD25+ Foxp3+ regulatory Tregs induced by various mechanism, enable to counteract the antitumor CTL response, resulting in the poor immunogens and immunosurveillance escape of tumor cells (Sakaguchi, 2005; Vignali et al., 2008). It seems to be a good strategy to exert antitumor effect by down-regulation of Tregs. Studies showed that BLP, a synthetic bacterial lipoprotein, can depress Foxp3 expression to abrogate Treg function via TLR1/2 signaling pathway, and thus up-regulate of CTLs (Zhang et al., 2011). In addition, DCs can also directly block the suppressive effect of CD4+, CD25+, and Treg cells, depending in part on IL-6 via TLRs signaling pathway (Pasare and Medzhitov, 2003). Of note, the process of T cell response mediated TLRs signaling pathway is showed in **Figure 1**.

### TLRs Agonists Act as Immune Adjuvant in the Cancer Therapy

Approaches that aiming at co-activated pathways are widely developed. Treatment upon some cancer by combination of TLRs agonists with both radiotherapy and bio-chemotherapy, are suggested to exhibit positive immunologic effect and reduce immune tolerance. Evident also showed that TLR-based adjuvant has generated preferable efficacy and clinical responses for the cancer patients in some extend (He et al., 2015; Mahoney et al., 2015).

Radiotherapy is still the main non-surgical treatments for most cancer patients in the worldwide. It induced a lethal DNA damage, leading to the cellular death and mitotic catastrophe blocking (Delaney et al., 2005). Nevertheless, radiotherapy is insufficient to elicit adequate immune response to control the tumor in many of cases, other measures must be involved in (Formenti and Demaria, 2013; Schölch et al., 2015). TLRs agonists are proposed to be preferable adjuvant in many researches. It has demonstrated that systemically administrated TLR 4 agonists, potentiates the effect of radiotherapy in murine solid tumor with greater frequency of CTLs (Apetoh et al., 2007). Sandra et al. have also suggested that TLR7 agonist IMQ (**Table 1**) acting as adjuvant when combined with radiotherapy, can increase cytokine IL-10 expression and elicit T cell response, exhibiting good prognosis among breast cancer patients in the phase I/II clinical trial (Demaria et al., 2013). In addition, damage of radiation exposure from cancer radiotherapy is unavoidable (Singh et al., 2015). Of note, activation of TLRs can also contribute to hematopoietic replenishment as its up-regulation of growth factor G-CSF, alleviating the intrinsic repair to radiant injuries (Singh et al., 2012; Kurkjian et al., 2017).

On the other hand, TLRs agonists can resist the tolerance in some of the cancers with combination of bio-chemotherapy. For example, Bacillus Calmette-Guerin (BCG), a ligand of TLR2 and TLR4, was used in the treatment of high-risk non-muscle-invasive bladder cancer against mycobacterium tuberculosis. However, antimicrobial peptides (AMPs) and massive pro-inflammatory cytokines were releases as the activation of TLRs via nuclear factor-κB (NF-κB) and mitogen activated protein kinases (MAPK) pathway, resulting in the resistance toward mycobacteria (Akira et al., 2001; Ryffel et al., 2005). Evident showed that Poly (I:C) (**Table 1**) can induce the secretion of MHC class I molecules via TLR3 signaling pathway, increasing the tumor immunogenicity to overcome this resistance (Ayari et al., 2016). In addition, TLRs agonists can also elevate the efficacy in some of vaccine design. NY-ESO-1 is considered as a safe and compatible tumor antigen in design of vaccine, due to its extensive presence in various tumor types and the strong spontaneous cellular immune responses including activation of CD4 and CD8+ T cells in the vitro and vivo study (Ayari et al., 2016). However, it is a pity that faint tumor regression is observed on cancer patients in the clinic research. Nina et al. has found that NY-ESO-1 in combination with a TLR7/8 agonist resiquimod (**Table 1**), can develop notable anti-NY-ESO-1 IgG antibody titers and enhanced T cells response in all high-risk melanoma patients, showing a predominant adjuvant effect of resiquimod in cancer treatment (Adams et al., 2008; Lubong et al., 2015). Altogether, these data show that activation of TLRs play an important role in the immune response, exhibiting particular prospect of TLRs agonists in the various cancer immunotherapy by acting as an adjuvant. It is worth to further investigate the adjuvant effect of TLRs agonists and achieve preferable vaccine application on the therapy of cancers and other diseases. The recently ongoing clinical studies of TLRs agonists in cancer treatment combined with other therapies are showed in **Table 1**.

## Negative Effect of TLRs in Tumor Progression

Although TLRs have generated significant efficacy in the cancer therapeutic studies and even in the clinical phages (Galluzzi et al., 2012), various scientists still query the effect as the existing studies show tumor progression, metastasis and recurrence in cancer therapy with the activation of TLRs (Grimmig et al., 2016; Ntoufa et al., 2016; Zhang et al., 2017). Tumor environment is a complex constitution of immune suppressive cells (Sato et al., 2009). However, TLRs can express not only in immune cells but also in this kind of tumor-associated cells and lead to tumor exacerbation. For example, the recurrent esophageal squamous cell carcinoma (EC) tissue exhibited significant higher expression of TLR3 and TLR4, which is associated with poor prognosis and a high probability of lymph node metastasis (Sheyhidin et al., 2011). In addition, cancer cells enable activate personal TLRs signals to release cytokines and chemokines in the tumor environment, which in turn to bind the immune suppressive cells and further release aberrant cytokines and chemokines, ultimately leading to tumor progression. Cancer cells from 133 prostate cancer patients have showed higher TLR3 and TLR9 expression accompanied with aberrant level of cytokines, inhibiting cell cycle induced apoptosis (González-Reyes et al., 2011). On the other hand, Liu et al. have also demonstrated that the tumor-derived snRNA-rich exosomes, can move to lung epithelial cells and activate the TLR3 to elicit multiple inflammatory cytokines release, resulting in the recruitment of neutrophils in second site to create the adaptive environment for primary tumor to metastasis (Liu et al., 2016). It shows that complicated interaction between tumor cells and immune cells in the tumor environment contributes to the aberrant immune enhancement and promote anti-tumor effect in TLRs signaling pathway. This can neatly illustrate the poor efficacy of some TLR agonists in the tumor clinical treatment. At present, the dual effects of TLRs regulators on tumors block the clinical practice. More and more studied are under intensively conducted for a systematical theory. Some of effective developed options are mentioned to solve this problem, such as targeted drug use and combination therapy, showing great advantages in clinical medication.

## Conclusion

As the significant PRR, TLRs can defense host from invading pathogen, cancer cells and aberrant product by eliciting innate and adaptive immune response. In this review, we have highlighted the activation of TLRs in different phenotypes of cells that have the potential to stimulate immune response and PCD process to cancer cells. Although evident shows the contribution of TLRs to cancer treatment has generate a great effect, the pursuit to achieve an excellent treatment toward various tumors is still rough as the complexities and uncertainty of tumor environment. It remains effort to explore a bright and effective modality of tumor therapy in the future, and a better integration of different strategy upon cancer to achieve the maximum therapeutic efficacy. A fully exploit of the TLRs mechanism and modulators against tumor are desired and contributes to a better treatment of high-efficient, low-toxic, and well-tolerant to most cancer patients.

## Author Contributions

XC drafted the manuscript. KC and SL designed the project and revised the manuscript. All authors read and approved the final manuscript.

## Conflict of Interest Statement

The authors declare that the research was conducted in the absence of any commercial or financial relationships that could be construed as a potential conflict of interest. The reviewer XL and handling editor declared their shared affiliation.
